# Supplementary material for: Characterizing the Impact of Communication on Cellular and Collective Behavior Using a Three-Dimensional Multiscale Cellular Model
Source: Entropy (Basel). 2023 Feb 9;25(2):319. doi: 10.3390/e25020319 (PMC9955575; doi:10.3390/e25020319)
Supplement: Supplementary file 1 [file entropy-25-00319-s001.zip › entropy-2140008-supplementary.pdf]

Supplementary Materials: Characterizing the Impact of Communication on Cellular and Collective Behavior Using a Three-Dimensional Multiscale Cellular Model

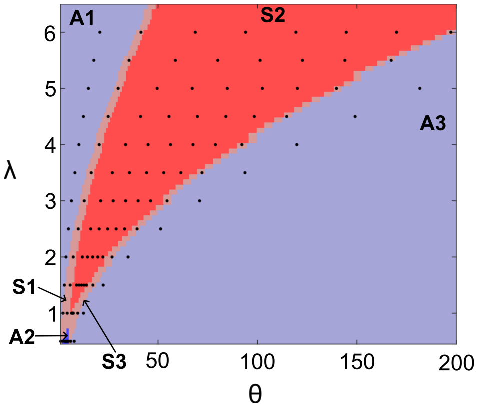

Figure S1. All sampled λ, θ values (black circles). For each of 12 λ values, 9 θ values were chosen—1 in region A1 (light blue); 1 in S1 (light red); 5 in S2 (dark red) or A2 (dark blue); 1 in S3 (light red); and 1 in A3 (light blue).

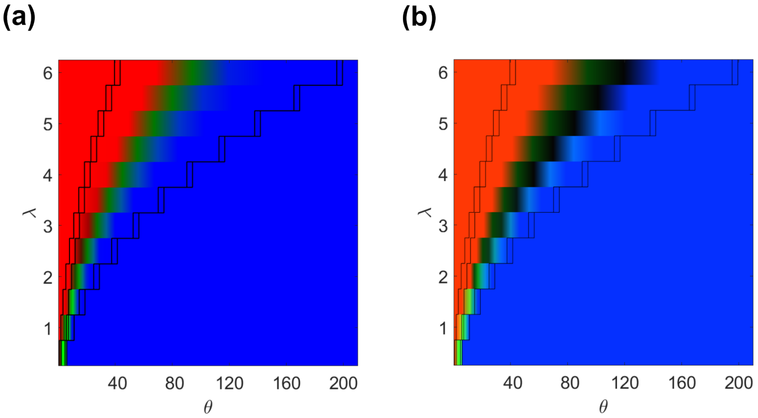

Figure S2. Example tissues in which asocial behavior is exhibited in social regions. (**a**) $D_H(A^{T_{\lambda,\theta}}, A^{T_{\lambda^{ON},\theta^{ON}}})$,$D_H(A^{T_{\lambda,\theta}}, A^{T_{\lambda^{OFF},\theta^{OFF}}})$, and $D_H(A^{T_{\lambda,\theta}}, A^{T_{\lambda^{SELF},\theta^{SELF}}})$ for a single tissue. The color is generated using $1 - D_H(A^{T_{\lambda,\theta}}, A^{T_{\lambda^{ON},\theta^{ON}}})$, $1D_H(A^{T_{\lambda,\theta}}, A^{T_{\lambda^{SELF},\theta^{SELF}}})$,$1D_H(A^{T_{\lambda,\theta}}, A^{T_{\lambda^{OFF},\theta^{OFF}}})$ as (R,G,B) values. In bright regions, $b_{\lambda,\theta}$ is low and tissues behave similarly to in the asocial regions—color indicates the asocial region. In dark regions, $b_{\lambda,\theta}$ is high and tissues behave differently than in asocial regions. $b_{\lambda,\theta} = 0$ in regions S1, S3 and on the left and right edges of S2. Cells on the left edge behave the same as in the ON region, while cells on the right edge behave the same as in the OFF region. (**b**) $D_{KL}(D_{\lambda,\theta}, D_{\lambda^{ON},\theta^{ON}})$, $D_{KL}(D_{\lambda,\theta}, D_{\lambda^{OFF},\theta^{OFF}})$, $D_{KL}(D_{\lambda,\theta}, D_{\lambda^{SELF},\theta^{SELF}})$ for a single tissue. The color is generated using $1D_{KL}(D_{\lambda,\theta}, D_{\lambda^{ON},\theta^{ON}})$, $1D_{KL}(D_{\lambda,\theta}, D_{\lambda^{SELF},\theta^{SELF}})$, $1D_{KL}(D_{\lambda,\theta}, D_{\lambda^{OFF},\theta^{OFF}})$ as (R,G,B) values. In bright regions, $\hat{D}_{KL}(\lambda, \theta)$ is low and tissues behave similarly to in the asocial regions—color indicates the asocial region. In dark regions, $\hat{D}_{KL}(\lambda, \theta)$ is high and tissues behave differently than in asocial regions. $\hat{D}_{KL}(\lambda, \theta) = 0$ within S1, S2, and S3. Tissue samples in S1 and on the left edge of S2 have the same composition as in the ON region and tissue samples in S3 and right edge of S2 have the same composition as in the OFF region.

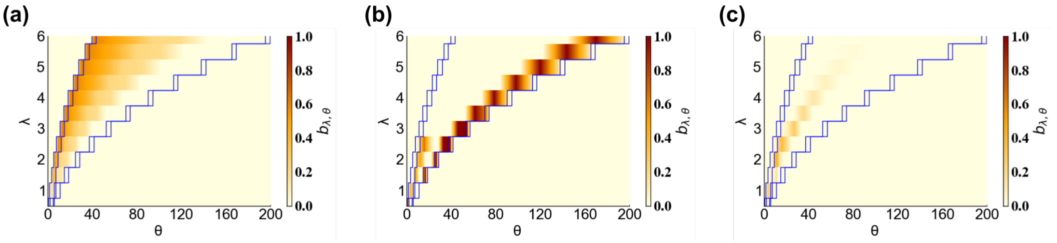

Figure S3. Three examples of variable behavior of $b_{\lambda,\theta}$ in different tissues; (**a**) a very wide single peak; (**b**) a double peak in which one peak terminates as λ increases; (**c**) a single peak that fades as λ increases.

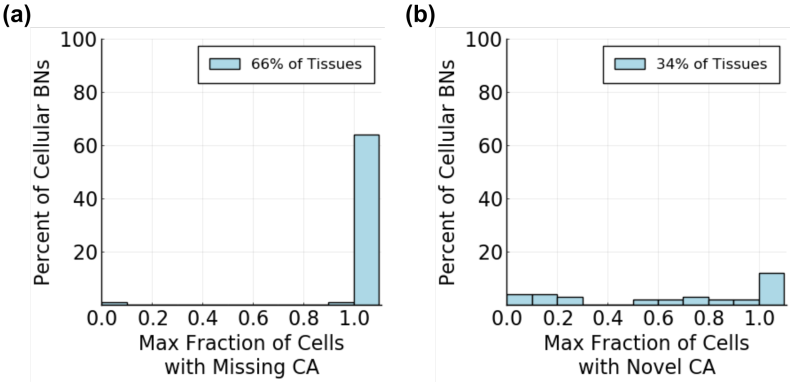

Figure S4. Frequencies of observing Lost or Novel CAs in tissues. To check that the missing or novel CAs are not infrequent cellular behaviors, and therefore less likely to be biologically meaningful, we measured their frequencies within a given tissue sample, reporting the maximum across all communication parameter values (**a**) distribution of the maximum frequency of Lost CAs in an asocial tissue sample; (**b**) distribution of the maximum frequency of Novel CAs in a social tissue sample.

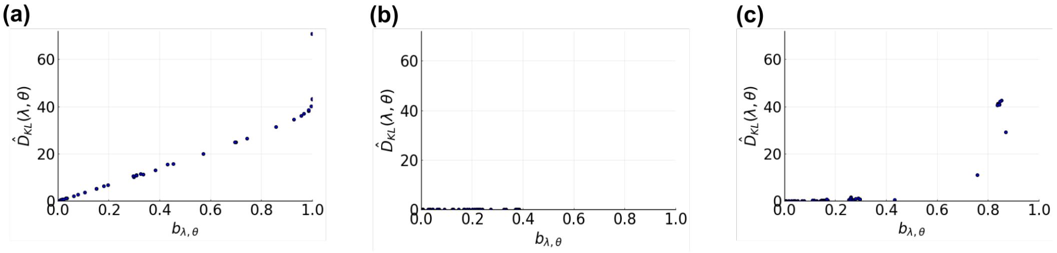

Figure S5. Three tissue examples of how changes in cellular behavior, $b_{\lambda,\theta}$ can relate to changes in tissue composition, $\hat{D}_{KL}(\lambda,\theta)$. Each data point represents the $b_{\lambda,\theta}$ and $\hat{D}_{KL}(\lambda,\theta)$ of a single tissue sample. (**a**) A tissue with a strong positive relationship between $b_{\lambda,\theta}$ and $\hat{D}_{KL}(\lambda,\theta)$; (**b**) a tissue with a very weak positive relationship between $b_{\lambda,\theta}$ and $\hat{D}_{KL}(\lambda,\theta)$; (**c**) a tissue with a switch-like relationship between $b_{\lambda,\theta}$ and $\hat{D}_{KL}(\lambda,\theta)$.

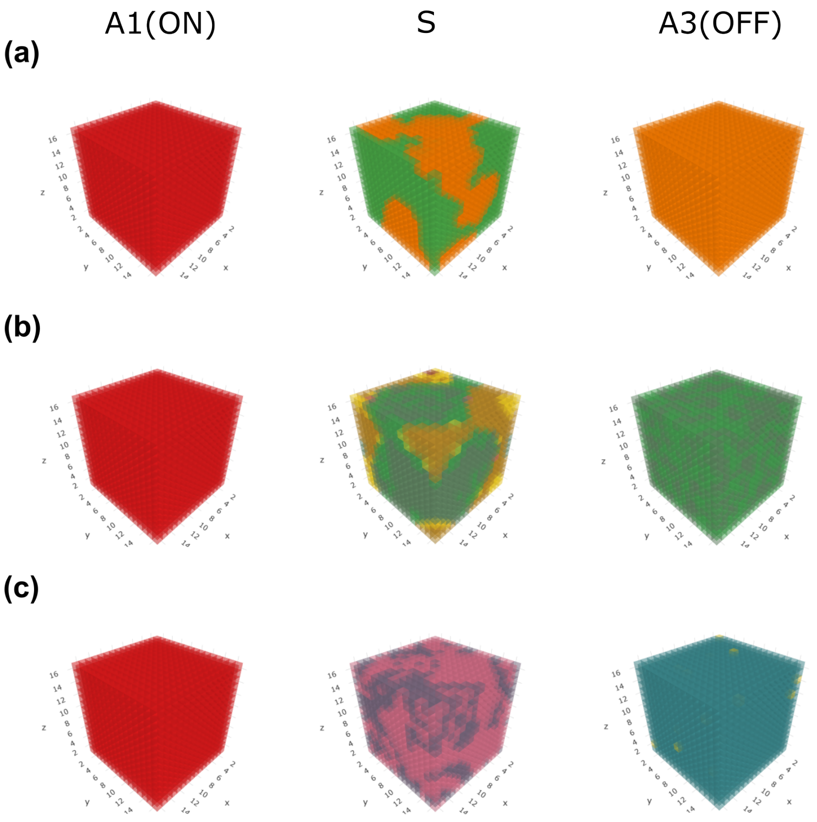

Figure S6. Three tissue examples of spatial organization occurring within the social regions of the parameter space. Tissue samples from the A1(ON), S1/S2/S3, and A3(OFF) regions are shown from each tissue. Each subplot visualizes the $16 \times 16 \times 16$ grid of cells(small cubes) colored by the CA it occupies. CA colors are consistent within a tissue but not preserved in different tissues. Cells are slightly transparent so inner layers of the tissue sample can be seen. (**a**) a tissue with patterning of 2 CAs in S3, 1 (orange) common with the OFF region and 1 (green) with the SELF region (not shown); (**b**) a tissue with patterning of 8 CAs in S2, 2 (green,purple) common with the OFF region and 6 unique to social regions; (**c**) a tissue with patterning of 2 CAs in S1, 1(purple) common with the SELF region (not shown) and 1 unique to social regions.
